# Modified Conservative Technique of Odontogenic Keratocyst Treatment With Long-Term Follow-Up: A Case Report

**DOI:** 10.7759/cureus.77010

**Published:** 2025-01-06

**Authors:** Mohammed M Al-Ali, Amjad R Al-Ateyah, Ali H Al-Thani

**Affiliations:** 1 Oral and Maxillofacial Surgery, King Fahad General Hospital, Hofuf, SAU

**Keywords:** conservative management, conservative treatment, custom-made, keratocyst, odontogenic keratocyst (okc), soft tray

## Abstract

An odontogenic keratocyst is a benign developmental cyst that affects the jawbone and has aggressive characteristics. Compared to other odontogenic cysts, it has a high recurrence rate. Standardized treatment protocols for this cyst remain a topic of debate. This case report describes a 42-year-old Saudi male patient with a recurrent odontogenic keratocyst involving a mandibular right angle. The patient underwent conservative treatment involving a modified technique utilizing a specially designed, custom-made soft tray to keep the surgical site open for potential re-curettage, aimed at reducing the chances of recurrence. The cystic lesion was effectively and successfully treated with no recurrence observed throughout the six-year follow-up period, emphasizing the importance of close monitoring and follow-up in managing odontogenic keratocysts.

## Introduction

An odontogenic keratocyst (OKC) is a benign developmental odontogenic abnormality with aggressive characteristics affecting the jaw bone derived from the remnants of the original tooth germ or dental lamina [1].

In 2005, the World Health Organization (WHO) altered the classification of OKC by categorizing them as tumors in the updated versions of its classification system [2]. The recommended term became keratocystic odontogenic tumor (KCOT). Nonetheless, in 2017, the WHO reverted the KCOT classification to OKC and remained OKC in the last edition of its classification of odontogenic tumors [3,4].

OKCs can develop across different age ranges, yet they are most commonly found in younger individuals, particularly during the second to fourth decades of life. These tumors typically involve the posterior body and ascending ramus of the mandible [1,4].

OKCs are frequently asymptomatic and are usually discovered incidentally during routine dental examinations or on radiographs taken for other reasons. A notable characteristic of OKCs is their tendency to recur after being surgically treated, which makes them more aggressive than other odontogenic cysts. The presence of multiple OKCs is commonly linked with nevoid basal cell carcinoma syndrome (Gorlin-Goltz syndrome) [1,5].

The management of OKC remains a subject of debate due to the variety of approaches available, each with its benefits and drawbacks. These strategies range from conservative options like enucleation with primary closure or open packing, decompression, or marsupialization to more aggressive methods involving radical surgical techniques and bone resection [1,5,6].

In this report, we presented a case of recurrent OKC that had been successfully treated using a specially designed, custom-made soft tray. We aimed to emphasize the efficacy of a conservative treatment strategy for OKC and the importance of long-term follow-up.

## Case presentation

A Saudi male patient, aged 42 years, was referred to our oral and maxillofacial surgery clinics in October 2018 for the evaluation and additional assessment of a radiolucency identified in the right posterior area of the mandible during a regular dental check-up. He had no significant medical history. In dental history, the patient described a prior lesion in the exact location around two years ago that was excised while the patient was under general anesthesia. The extra-oral and intra-oral examinations revealed no swelling or color changes, and the patient reported no symptoms.

**Figure 1:**
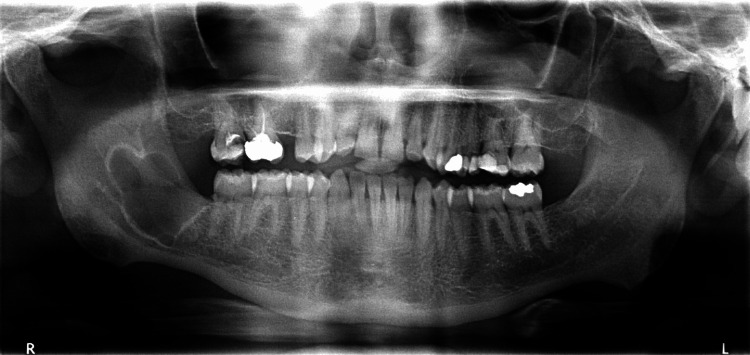
Radiographic imaging The orthopantomogram showed a well-circumscribed radiolucent lesion in the right posterior mandible.

Radiographic examination displayed a well-circumscribed radiolucency involving the right posterior area of the mandible with a border characterized by scalloping and sclerosis. The lesion extends from the lower right second molar (distal surface) to nearly the middle of the ramus without impacting neighboring structures. It measured about 3.5×2.5 cm (Figure 1).

After reviewing the radiographs, the potential diagnoses we considered included residual cyst, OKC, and unicystic ameloblastoma.

**Figure 2:**
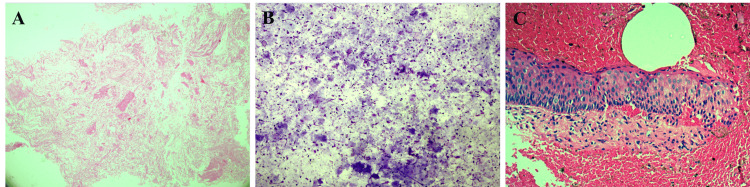
Microscopic examination A fine needle aspiration cytology smear shows corrugated keratinous material (A), with a few nucleated and anucleated squamous cells in a background of keratin debris (B). Hematoxylin and eosin-stained microscopic photograph of the incisional biopsy shows corrugated parakeratinized stratified squamous epithelium of uniform thickness with palisaded hyperchromatic basal cell layer and flat junction between the epithelium and connective tissue (C).

A straw-colored and cheesy fluid was obtained through fine needle aspiration. Subsequently, an incisional biopsy was conducted, placed in 10% neutral buffered formalin, and sent for histopathological examination, revealing a cystic lesion lined by a consistently thick layer of stratified squamous epithelium with corrugated and parakeratinized luminal cells. This epithelium displayed hyperchromatic and palisaded basal cells with an even junction between the epithelium and connective tissue (Figure 2A-2C).

With these findings, the final diagnosis was OKC.

After a discussion regarding treatment options, the patient opted for conservative treatment and refused a more aggressive approach under general anesthesia. As a result, an alternative conservative approach was proposed, involving the use of a specially designed custom-made soft tray to maintain the surgical site accessible for further curettage if needed, aiming to reduce the chances of recurrence.

**Figure 3:**
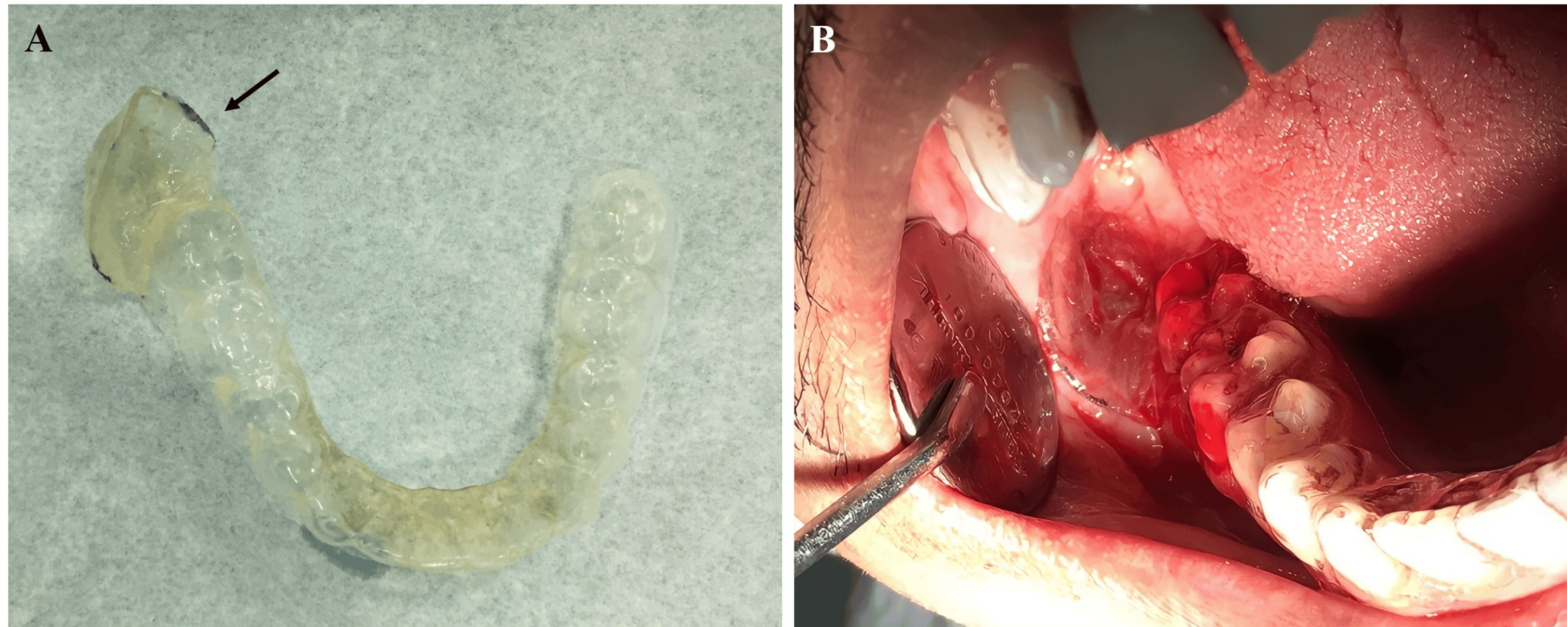
Specially designed custom-made soft tray The customized soft tray and strengthened posterior segment (arrow) (A). The customized soft tray is inserted and adapted inside the patient's mouth (B).

Following the patient's consent, the cyst was managed by deroofing, enucleation, and curettage on the chairside using lidocaine 2% local anesthetic agent with epinephrine vasoconstrictor 1:80000. The excised specimen was taken for microscopic analysis. Concurrently, an impression was obtained using an alginate material to create a removable custom-made tray, which was taken to the laboratory, where a study cast was created. Subsequently, a customized soft tray was crafted on the study cast using a suck-down machine and ethylene vinyl acetate soft sheet. The posterior segment of the soft tray, designed to fit into the surgical site, was strengthened using self-curing orthodontic polymethyl methacrylate. After about half an hour, we fitted and modified the tray on the chairside and provided it to the recipient (Figure 3A, 3B).

**Figure 4:**
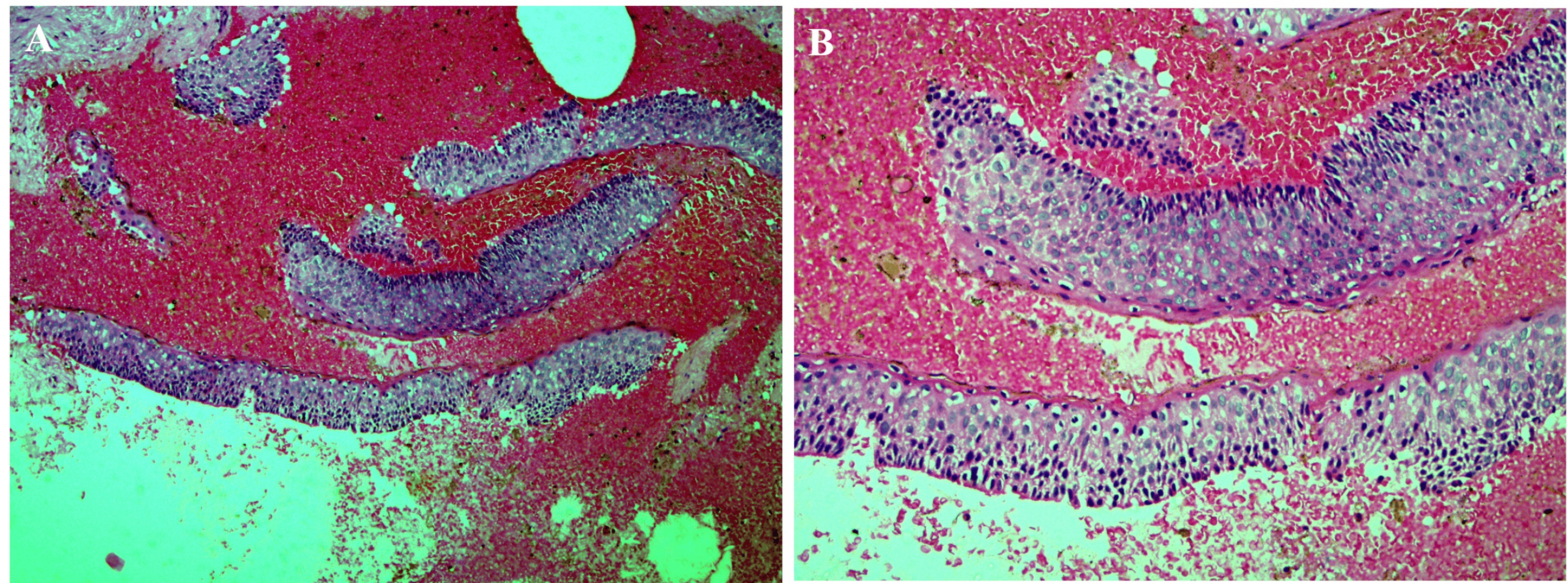
Hematoxylin and eosin-stained microscopic photographs of the excisional biopsy At low magnification (A) and higher magnification (B), multiple fragments of cystic lining characterized by a uniform thickness of corrugated parakeratinized stratified squamous epithelium and a hyperchromatic and slightly hyperplastic palisaded basal layer are seen.

The histopathological analysis of the excised tissue aligned with the initial biopsy findings, confirming the diagnosis of OKC (Figure 4A, 4B).

**Figure 5:**
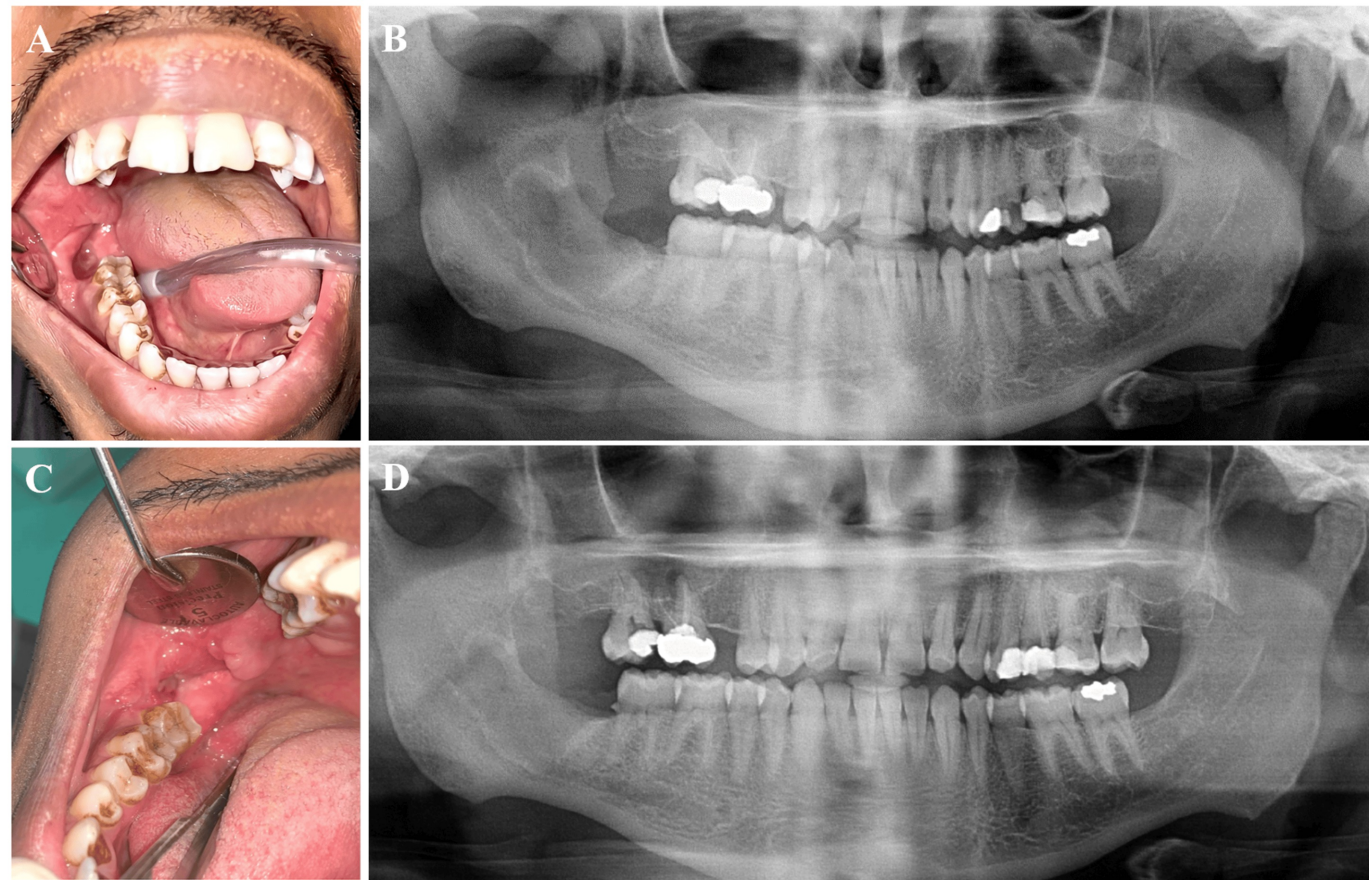
Postoperative intra-oral photographs and panoramic radiographs Two weeks of follow-up showed a clean surgical site with good initial healing (A and B). Two months of follow-up showed significant surgical site healing (C and D).

The patient reported that he found the tray comfortable, which did not impede his daily activities. He was able to maintain good oral hygiene and did not experience any unpleasant odor. Two weeks later, the lesion underwent re-curettage, and the extracted tissue was submitted for microscopic analysis. It showed no signs of OKC, and it was exclusively granulation tissue. A follow-up after two weeks displayed a well-healed surgical site, while a two-month follow-up illustrated significant healing progress (Figure 5A-5D).

**Figure 6:**
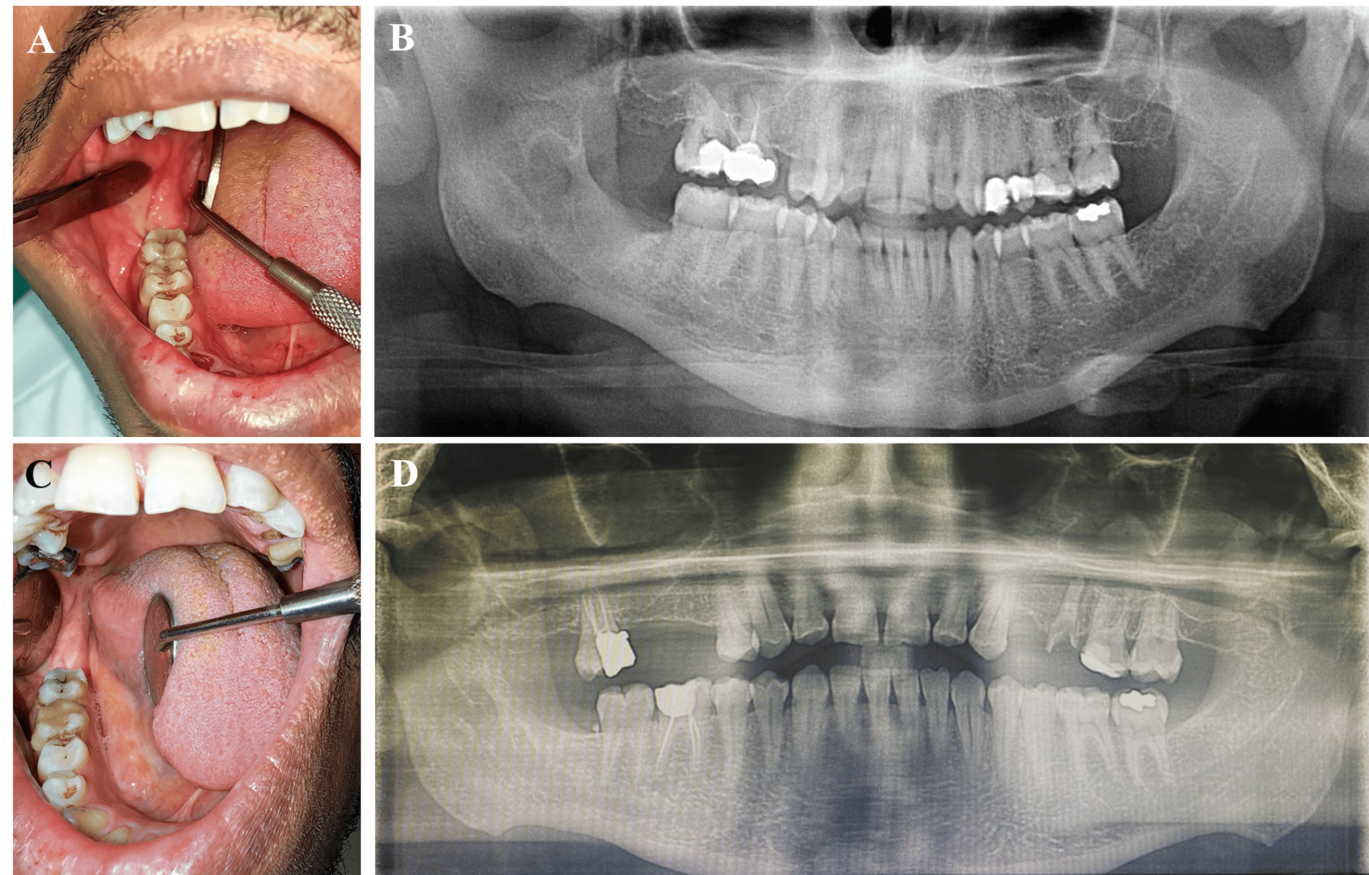
Postoperative intra-oral photographs and panoramic radiographs Four months of follow-up showed complete surgical site healing (A and B), and six years of follow-up showed no signs of recurrence (C and D).

During this visit, the patient was informed to discontinue using the tray. A four-month intra-oral examination demonstrated full recovery of the surgical area. The patient was monitored for more than six years with no signs of recurrence (Figure 6A-6D).

## Discussion

Following Philipsen's description of the term "keratocyst" for the first time in 1956, a significant amount of literature has emerged on this topic, discussing its etiology, development, clinical presentations, and diverse treatment approaches [7].

Identifying this cyst is essential because the OKC demonstrates a more aggressive nature and exhibits a higher recurrence rate than other odontogenic cysts; most recurrences typically occur within the initial five years following the first treatment. Additionally, it may be associated with Gorlin-Goltz syndrome [8,9]. Several factors contribute to the recurrence rate of OKC, including a thin and friable wall that can easily detach, the existence of satellite and daughter cysts, and their aggressive nature that tends to infiltrate surrounding tissues, potentially causing incomplete removal and subsequent recurrences. The choice of surgical technique also plays a significant role in this context [9-11].

There needs to be more consensus in the literature concerning a standardized treatment protocol for OKCs, which varies from less invasive to more aggressive surgical methods, with insufficient evidence to definitively identify the most effective approach in reducing morbidity or preventing recurrence [1,5,12]. Nevertheless, the reclassification of OKCs within the odontogenic cysts category and the benign character of the lesion have encouraged a shift towards conservative techniques that aim to minimize morbidity, thus establishing a conservative approach as the preferred technique for surgeons [12,13].

The utilization of decompression in conjunction with enucleation is a beneficial treatment choice, especially when combined with peripheral osteotomy. This combined method has demonstrated a significant reduction in the recurrence rate and is endorsed in the recommended treatment protocol for OKCs [13,14].

Decompressing's advantage lies in its ability to decrease intra-cystic pressure by draining cystic fluid. This process assists in reducing the lesion's size, consequently reducing the harm to adjacent structures, enhancing surgical access and effectiveness, and fostering a conducive environment for healing and generating new bone around the cystic cavity as the lesion progressively collapses [14].

Scientific literature has documented numerous methods for conducting cystic decompression, such as gauze packing the cystic cavity, suturing a device to its edge, or using an acrylic stent with a clasp (denture base). These methods' primary reported drawbacks include infection, patient discomfort, food accumulation and unpleasant odors, tissue injury, potential tube or stent blockages, and the risk of device dislodgment, displacement, and lesion recurrence [15-17].

Due to the shrinkage and new bone formation promoted by the decompression of the odontogenic cyst and the subsequent reduction in cyst size, the lesion is effectively pushed upward, facilitating its surgical removal [13-17]. Our technique effectively maintained an open surgical site, enabling re-curettage. We opted for re-curettage and sent the excised tissue for histopathological examination to eliminate any remnants of cystic tissue, thereby reducing the risk of OKC recurrence.

In our case, the patient expressed significant comfort with the tray, finding it easy to maintain cleanliness from food and debris, which helps avoid site infection. Additionally, we observed no inflammation or injury to adjacent tissues, and there was no presence of foul odor. Notably, the operative site in this case entirely healed within four months, demonstrating a quicker recovery period than the reported average means of decompression time, ranging from 8.4 to 17.5 months, and the average means of reduction rate, ranging from 65% to 81% [17].

To the best of our knowledge, no reported instances of utilizing this specific technique for managing an OKC are in the literature. The case presented was managed conservatively and adhered entirely to established treatment guidelines recognized by the medical community for such scenarios, using a modified approach involving a custom-made tray, resulting in the successful treatment of the lesion without recurrence during the six-year follow-up period.

## Conclusions

Considering the patient's comfort, the expected reduction in infection risk, and the ease of performing multiple curettages with minimal disturbance to both the patient and the surgical site to lower the chances of recurrence, we highly endorse implementing our proposed approach for OKC conservative management and similar oral lesions, where non-invasive management and decompression are encouraged, with close and thorough follow-up to ensure long-term success.
